# Dynamic causal modeling analysis reveals the modulation of motor cortex and integration in superior temporal gyrus during multisensory speech perception

**DOI:** 10.1007/s11571-023-09945-z

**Published:** 2023-03-04

**Authors:** Ting Zou, Liyuan Li, Xinju Huang, Chijun Deng, Xuyang Wang, Qing Gao, Huafu Chen, Rong Li

**Affiliations:** The Clinical Hospital of Chengdu Brain Science Institute, MOE Key Laboratory for Neuroinformation, High-Field Magnetic Resonance Brain Imaging Key Laboratory of Sichuan Province, School of Life Science and Technology, University of Electronic Science and Technology of China, Chengdu, 610054 People’s Republic of China

**Keywords:** Dynamic causal modeling, McGurk effect, Multisensory information processing, Superior temporal gyrus, Motor cortex

## Abstract

The processing of speech information from various sensory modalities is crucial for human communication. Both left posterior superior temporal gyrus (pSTG) and motor cortex importantly involve in the multisensory speech perception. However, the dynamic integration of primary sensory regions to pSTG and the motor cortex remain unclear. Here, we implemented a behavioral experiment of classical McGurk effect paradigm and acquired the task functional magnetic resonance imaging (fMRI) data during synchronized audiovisual syllabic perception from 63 normal adults. We conducted dynamic causal modeling (DCM) analysis to explore the cross-modal interactions among the left pSTG, left precentral gyrus (PrG), left middle superior temporal gyrus (mSTG), and left fusiform gyrus (FuG). Bayesian model selection favored a winning model that included modulations of connections to PrG (mSTG → PrG, FuG → PrG), from PrG (PrG → mSTG, PrG → FuG), and to pSTG (mSTG → pSTG, FuG → pSTG). Moreover, the coupling strength of the above connections correlated with behavioral McGurk susceptibility. In addition, significant differences were found in the coupling strength of these connections between strong and weak McGurk perceivers. Strong perceivers modulated less inhibitory visual influence, allowed less excitatory auditory information flowing into PrG, but integrated more audiovisual information in pSTG. Taken together, our findings show that the PrG and pSTG interact dynamically with primary cortices during audiovisual speech, and support the motor cortex plays a specifically functional role in modulating the gain and salience between auditory and visual modalities.

**Supplementary Information:**

The online version contains supplementary material available at 10.1007/s11571-023-09945-z.

## Introduction

Humans communicate everyday by processing multimodal sensory information, especially integrating the auditory and visual inputs. The McGurk effect is well known in the multisensory integration underlying human speech perception. Listeners often perceive a McGurk illusion when incongruent auditory and visual signals are combined together (McGurk and MacDonald 1976). When an auditory syllable /pa/ was presented with a visual syllable /ka/, a different auditory syllable /ta/ will be perceived. Although the McGurk effect is robust and prevalent, the susceptibility of illusion varies among different individuals (Barutchu et al. 2019; Feng et al. 2019; Mallick et al. 2015). The individual McGurk susceptibility is correlated with ability of multisensory integration (Beauchamp et al. 2010; Benoit et al. 2010; Marques et al. 2016; Nath and Beauchamp 2012) and the ability of coordinated audiovisual modulation (Li et al. 2021).

Investigating the neural substrates that contribute to the McGurk illusion is of importance in helping understand the mechanisms underlying the multisensory information processing. Previous studies have suggested that the parts of Wernicke’s area (BA 42/22) in the posterior superior temporal cortex, especially the superior temporal gyrus (STG) and sulcus are involved in the multisensory speech processing (Friederici et al. 2017; Friederici 2011). Sensitivity to different phonetic features has been demonstrated in the middle and posterior superior temporal gyrus (mSTG/ pSTG) by using data-mining algorithms to identify patterns of activity in functional magnetic resonance imaging (fMRI) (Campbell 2011; Kilian-Hütten et al. 2011). Functional brain neuroimaging studies have demonstrated that the posterior superior temporal gyrus/sulcus (pSTG/S) is critical in the multisensory integration of audiovisual speech information (Beauchamp 2016; Beauchamp et al. 2004, 2008; Park et al. 2018) and the processing of short-timescale patterns (i.e., phonemes)-related activation is in the left mid-superior temporal gyrus (mSTG) (DeWitt and Rauschecker 2012). In addition to STG, motor structures play an important role in the multisensory speech perception (Benoit et al. 2010; Callan et al. 2014; D'Ausilio et al. 2009; Liebenthal and Möttönen 2018; Pulvermuller et al. 2006; Wilson et al. 2004). A study, by combining the fMRI and transcranial magnetic stimulation (TMS), directly proves that the TMS of the motor cortex lip areas weakened the McGurk effect greatly, suggesting that the motor network contributes to the illusion avoidance in the multisensory speech processing (Murakami et al. 2018). Moreover, a recent study reveals the redundant and synergistic cross-modal interactions in the left pSTG/S and motor cortex, respectively, during audiovisual speech processing (Park et al. 2018). Therefore, multiple neural mechanisms may support the audiovisual speech perception (Meijer et al. 2019). However, these studies typically investigated the STG/S and motor cortex activity related to the audiovisual speech stimuli and failed to focus on the dynamic interaction between audiovisual stimuli and brain signals.

The dynamic causal modeling (DCM) has been extended for causal inferences about the mechanism for an experimental condition to modulate the connections in a hypothesized neuronal network (Stephan et al. 2007; Zhang and Du, 2022). Previously, DCM can be applied to acquire the fMRI data during the audiovisual speech perception. For instance, both intrinsic STG and left to right STG connections are crucial in identifying the self-voice error and sensorimotor integration (Parkinson et al. 2013). A DCM study has shown that bidirectional connection between premotor cortex and STS and that between planum temporal and premotor cortex are significant during the speech perception, supporting the involvement of premotor cortex (Osnes et al. 2011). Besides, a study about the STS effective connectivity signature has suggested that the integration outcome of audiovisual speech primarily depends on whether the STS converges onto a multimodal syllable representation (Bouton et al. 2020). In general, the available evidence suggests both the effects of indirect and bidirectional influences of the STG/S and motor cortex on sensory processes during speech perception. However, they failed to explore the different modulatory influences of the pSTG/S and motor cortex either on auditory and visual processing areas and the effects of brain connections on the McGurk effect task performance. Studying the potential distinct functional roles of pSTG/S and motor cortex in processing streams of face and voice speech information will substantially advance the distinct mechanisms to support the audiovisual speech comprehension.

In the present study, behavioral measures and fMRI data were combined to explore the dynamic interactions of motor cortex and pSTG with primary sensory inputs during the audiovisual speech perception. We hypothesized that the motor cortex and pSTG involve in the distinct processes to support multisensory speech perception. Based on our previous study that focused mainly on brain activation instead of connectivity (Li et al. 2021), four brain regions from the left cerebral hemisphere were chosen for the DCM models: precentral gyrus (PrG, motor cortex), pSTG (multisensory region), mSTG (primary auditory speech processing), and FuG (visual lip movements processing). Forty-four models were selected to verify the effectiveness of network connectivity by these four regions of interest (ROIs). Random-effects Bayesian Model Selection (BMS) and Model Averaging (BMA) were applied to confirm the model fitting the observed data best and to estimate the subject-specific connectivity parameters. Subsequently, we employed the Spearman correlation analysis to explore the relation of model parameters to individual behavioral performance of McGurk effect. Finally, we further explored the connection strength differences between strong and weak McGurk perceivers.

## Materials and methods

### Participants

Sixty-three healthy volunteers (27 females, 21.7 years old in average (18–28 years)) who completed both behavioral and fMRI experiment were included in the study, as described in our previous study (Li et al. 2021). All participants were Chinese native speakers and right-handed, had normal or corrected-to-normal vision, and no any hearing disorders or psychiatric illnesses. They all had written informed consents on participating and understanding all experiments. This study was approved by the ethical committee of School of Life Science and Technology at University of Electronic Science and Technology of China.

### Stimuli and procedure

The stimuli and procedure was described in (Li et al. 2021). In brief, a behavioral experiment outside scanning was conducted to examine individual McGurk susceptibility. A task fMRI experiment was performed to examine the brain activity and time course in response to non-McGurk congruent audiovisual syllables.

**Fig. 1:**
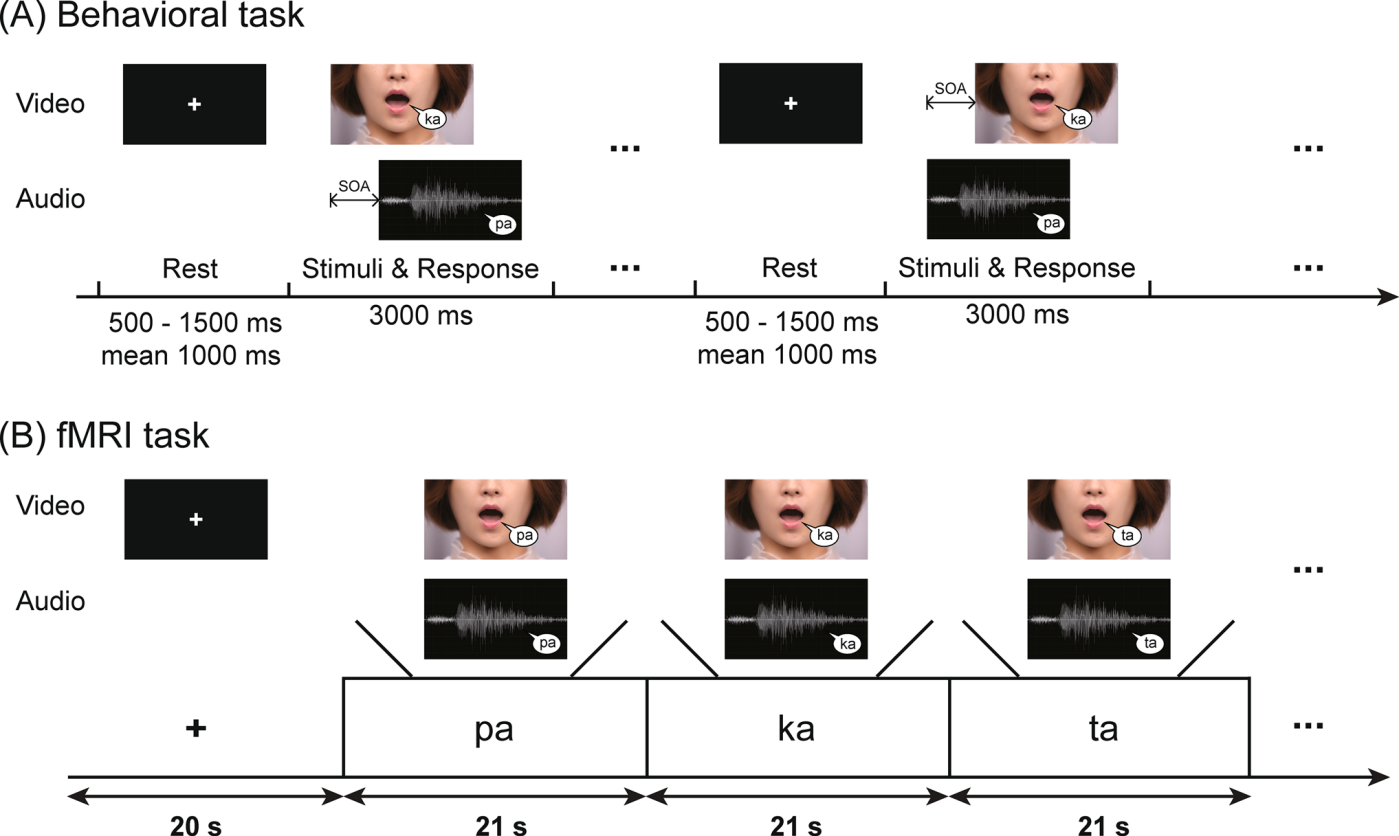
Behavioral and fMRI experimental paradigm. **A** In behavioral task, all participants were instructed to observe the lip movements and listen carefully, and then report what syllable they percept. **B** In the fMRI task, all participants were allowed to do the same actions with that in the behavioral task excluding reporting. SOA, stimulus onset asynchrony

In the behavioral McGurk experiment, we used the McGurk incongruent audiovisual syllable pairs consisted of an auditory recording of the syllable /pa/ and a digital video of a female speaker pronouncing the syllable [ka]. The combination of auditory track (/pa/) and visual track ([ka]) resulted in 29 stimuli onset asynchronies (SOAs), including 0 ms, ± 33 ms, ± 67 ms, ± 100 ms, ± 133 ms, ± 167 ms, ± 200 ms, ± 233 ms, ± 267 ms, ± 300 ms, ± 333 ms, ± 367 ms, ± 400 ms, ± 433 ms, and ± 467 ms (positive for visual-leading and negative for audio-leading). Stimuli for each SOA were presented 10 trails, and the 290 trails were randomly presented. When a trial was begun, a fixation cross was displayed at center of the visual field with jittered duration (random selection from 500/750/1000/1250/1500 ms), followed by stimuli presentation and response (3000 ms, Fig. 1A). The E-Prime version 2.0 (Psychology Software Tools, https://pstnet.com/) was employed for stimuli presentation. Visual stimuli were displayed by a Samsung Sync monitor with 1024 × 768 pixels, and participants were approximately 50 cm away. Auditory stimuli were given by a Sennheiser headphone at a comfortable and fixed level. Participants should fixate the center of monitor screen and to observe lip movements of the speaker. In addition, we informed participants to listen carefully and report the syllable that they perceived (/pa/, /ka/, or /ta/). Participants performed behavioral responses by pressing the corresponding keys on a standard terminal keyboard with their right hand.

The fMRI syllabic perception experiment was performed using the synchronous audiovisual-matched syllables, which included three conditions as follows: /pa/ + [pa], /ka/ + [ka], and /ta/ + [ta] (/auditory stimuli/ + [visual stimuli], Fig. 1B). Their order was balanced in three sessions (run1: /pa/, /ka/, and /ta/; run2: /ka/, /ta/, and /pa/; run3: /ta/, /pa/, and /ka/). A fixation cross was displayed at the visual field center with 20 s at the beginning of each session. After that, there were 18 blocks (6 blocks × 3 conditions) per session, and 7 trails were presented continuously in sequence in each block. Each trial consisted of audiovisual synchronous stimuli with 2000 ms and a fixation cross stimulus with 1000 ms. During fMRI data acquisition, a monitor viewed through a mirror equipped to the MRI head-coil was employed to present the visual stimuli and was about 60 cm from the participants. The projector was a 25.00 cm × 18.75 cm Avotec projector (SV6011) with 1024 × 768 pixels. Auditory stimuli were played using full-coverage Avotec headphones (SS3300) at 120 dB. Although the threshold of 120 dB marks the onset of pain during daily environment, the threshold was a comfort degree of our audio stimulus, allowing the participants (wearing earplugs and headphones) to hear the stimuli clearly under a noisy fMRI scanning environment. Participants were required to just observe the lip movements and listen carefully without reporting.

### fMRI data acquisition

All MRI data were collected using a 3.0 T GE 750 scanner (General Electric, Fairfield, Connecticut, USA) with high-speed gradients. Before scanning, participants were allowed to be familiar with the environment and sounds. The participants were required to wear eight-channel prototype quadrature birdcage head coils fitted with foam padding for the purpose of minimizing the head motion. Besides, the foam pads and earplugs were arranged for reducing the head movement and scanner noise to the maximal content. Functional images were captured by using a gradient-recalled echo-planar imaging (EPI) sequence. The repetition time = 2000 ms, echo time = 30 ms, flip angle = 90°, bandwidth = 250 Hz / pixel, 43 axial slices, slice thickness = 3.2 mm without gap, matrix = 64 × 64, and field of view (FOV) = 240 mm. Finally, we obtained 199 volumes for each participant.

### Behavioral data analysis

To quantify the individual McGurk susceptibility, we calculated the mean proportion of behavioral responses to 10 trails for 29 SOAs across all participants. When an auditory stimuli /pa/ and a visual stimuli [ka] were presented, participants may perceive McGurk fusion and report the syllable /ta/. The mean proportion of behavioral responses of /ta/ reached the peak at SOA of + 133 ms, so it was selected as an individual McGurk susceptibility for each participant (see (Li et al. 2021) for more details).

### fMRI preprocessing

In this present study, we selected the Pre-processing for Task fMRI Data module of Data Processing and Analysis of Brain Imaging (DPABI) version 6.1 (http://rfmri.org/dpabi) (Yan et al. 2016) to preprocess functional images. The first 10 functional images per session of each participant were discarded to ensure steady-state longitudinal magnetization. 189 remaining images of each session were slice-time corrected and realigned to the middle volume of every session for correction of inter-scan head motion. In addition, the mean frame-wise displacement (FD) was calculated for subsequent group comparisons (He et al. 2016; Li et al. 2018; Lu et al. 2020). Further, all images were spatially normalized to Montreal Neurological Institute (MNI) reference space by using EPI template and resampled to 3 × 3 × 3 mm^3^ voxels. The normalized functional images were finally smoothed with a full width half maximum (FWHM) Gaussian kernel of 6 mm.

### General linear model analysis

**Fig. 2:**
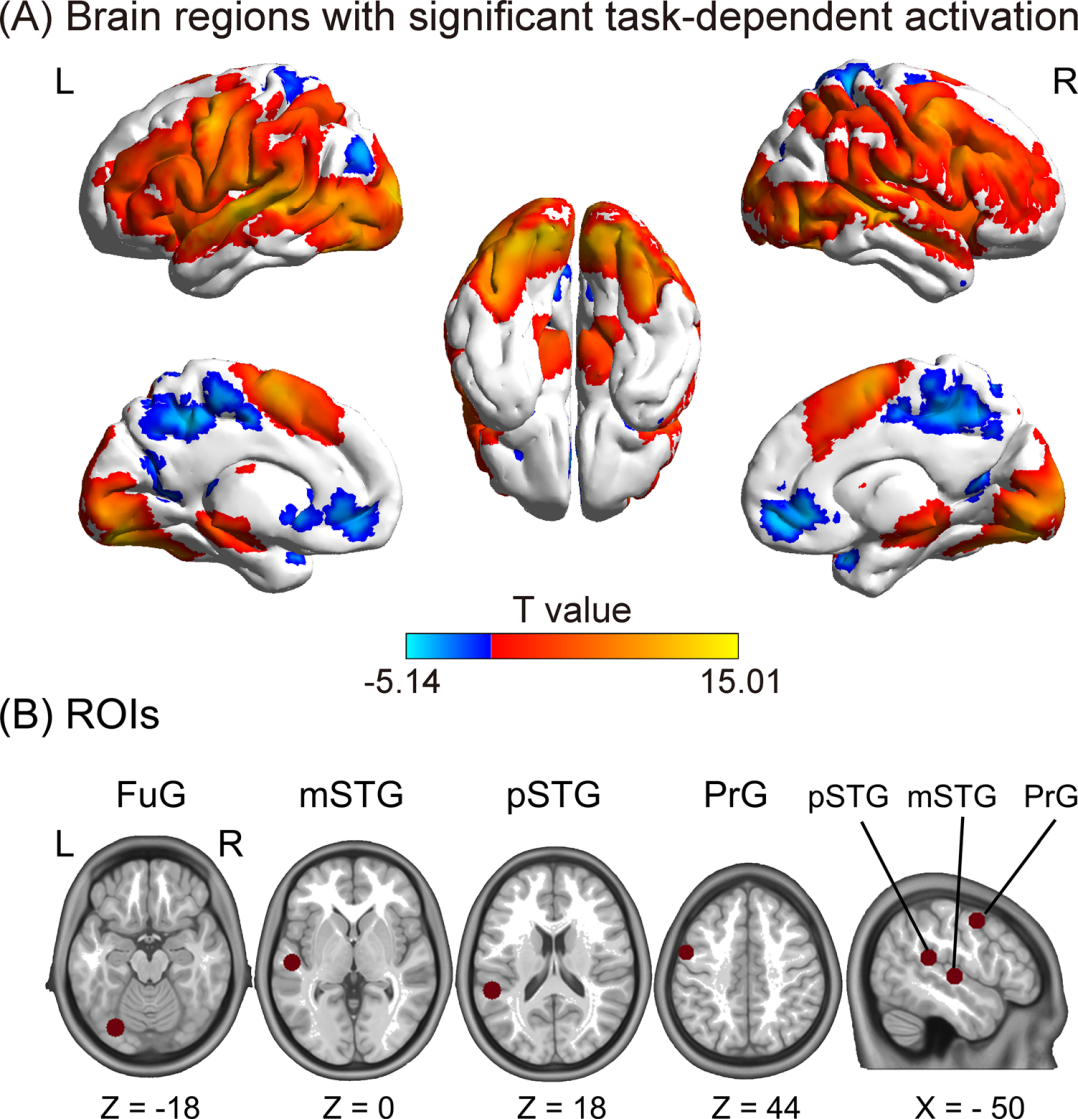
**A** Brain regions with significant task-dependent activation in the group-level statistic (*n* = 63; one-sample t-test, *P* < 0.05, FDR-corrected). Brain map was generated with BrainNet Viewer version 1.61 (http://www.nitrc.org/projects/bnv/); **B** ROIs. The anatomy map (*n* = 63) was employed to determine node peak selection, in which four peaks were showed in red. We only explored regions from the left hemisphere. The anatomy map was visualized with the MRIcron version 4 (https://www.nitrc.org/projects/mricron). MNI, Montreal Neurological Institute; FuG, fusiform gyrus; pSTG, posterior superior temporal gyrus; mSTG, mid-superior temporal gyrus; PrG, precentral gyrus

Task-dependent activation was estimated by performing a single-subject analysis with a general linear model (GLM) in Statistical Parametric Mapping (SPM) version 12 (release 6225, https://www.fil.ion.ucl.ac.uk/spm). Prior to this process, the analyzed data were high-pass filtered with a cut-off period of 128 s to exclude the slow signal drifts. In the present study, the data were modeled as a parametric design. Three syllable stimuli (/pa/ + [pa], /ka/ + [ka], and /ta/ + [ta]) were modeled together as a regressor according to the absence of significant main effect of three syllables (Li et al. 2021). We calculated six realignment parameters (three translations and three rotations) for each volume when the motion was corrected, and they were added as nuisance covariates. Each block was convolved by a canonical hemodynamic response function with a duration of 21 s and a stimulus onset interval of 21 s. A T-statistical contrast was specified for task-dependent effects. The finally obtained images were underwent a second-level analysis. One-sample *t* test was used to infer significantly different activations on the group-level (*n* = 63; *P* < 0.05, FDR-corrected, Fig. 2A). Furthermore, we specified an F-statistical contrast for subsequent volumes of interest (VOI) extraction when it was adjusted for effects of interest (Torrisi et al. 2013).

### Dynamic causal modeling analysis

DCM can be adopted to explain the changes in regional activity modulating the connectivity among different brain regions (Friston et al. 2003). In present study, DCM and BMA analyses were applied to investigate the dynamic interactions between unimodal and multimodal audiovisual systems during the perception of synchronized and matched audiovisual syllables, which were conducted within DCM12 as implemented in SPM12 (https://www.fil.ion.ucl.ac.uk/spm).

#### ROIs selection

We focused on two unimodal sensory areas for DCM, including the primary auditory and visual speech cortex and two multisensory speech processing areas. Previous studies have supported the role of the mSTG as primary auditory cortex (DeWitt and Rauschecker 2012; Friederici et al. 2017). Visual speech activations have been reported in the FuG (Bernstein and Liebenthal 2014; Campbell 2011; Capek et al. 2008). In terms of multisensory processing, it has been converged that the pSTG/S is very critical for the cross-modal integration of audiovisual speech information (Beauchamp 2016; Beauchamp et al. 2004, 2008; Park et al. 2018). In addition to incorporating these three brain regions into the DCM model, the motor cortex was also included as an important multisensory speech processing region (Benoit et al. 2010; Callan et al. 2014; D'Ausilio et al. 2009; Liebenthal and Möttönen 2018; Pulvermuller et al. 2006; Wilson et al. 2004). To be specific, as regions showing significantly different activations on the group-level (Table 1) as well as between strong and weak perceivers (Supplementary Table S1), these four ROIs were identified with peak MNI coordinates of group-level task activation maps: left PrG (motor cortex: − 54, 0, 48), left pSTG (multisensory region: − 54, − 39, 18), left mSTG (auditory speech processing: − 54, − 18, 3), and left FuG (visual lip movements processing: − 30, − 78, − 18) (Table 1 and Fig. 2B). Locations of the ROIs could be determined by limiting the four seed regions by an 8 mm sphere around the peak voxel (MNI coordinates of the highest T value). Subsequently, the ROIs were overlapped with the corresponding Automated Anatomical Labeling atlas (Tzourio-Mazoyer et al. 2002) to ensure the brain voxels within each ROI fell inside the corresponding specific brain regions.

**Table 1:** Brain regions with significant task-dependent activation

Cluster number	Brain regions	MNI coordinates (x, y, z)			Cluster size	Peak T value
Cluster 1	L mid-superior temporal gyrus	− 54	− 18	3	19377	15.01
	L Fusiform gyrus	− 30	− 78	− 18		11.68
	L Precentral gyrus	− 54	0	48		10.42
	L Posterior superior temporal gyrus	− 54	− 39	18		9.69
	L Postcentral gyrus	− 63	− 21	15		10.61
	L Middle frontal gyrus	− 39	51	12		5.26
	L Middle occipital gyrus	− 45	− 78	3		8.91
	L Cerebellum	− 45	− 57	− 27		10.77
	L Inferior parietal loulbe	− 27	− 57	48		4.61
	R Superior temporal gyrus	54	− 18	0		14.06
	R Middle temporal gyrus	48	− 63	0		10.77
	R Cerebellum	12	− 75	− 15		11.12
	R Precentral gyrus	51	3	48		10.38
	R Middle frontal gyrus	39	54	6		4.93
	R Anterior cingulate cortex	9	33	− 6		− 4.66
Cluster 2	L Supplementary motor area	0	3	63	1031	11.56
	R Supplementary motor area	6	9	69		7.64
Cluster 3	L Middle cingulate cortex	− 12	− 39	42	1842	− 5.14
	L Precuneus	− 12	− 42	45		− 4.79

The first four regions were selected for further analysis in our study (n = 63; *P* < 0.05, false discovery rate (FDR) corrected); MNI, Montreal Neurological Institute; X, Y, Z, coordinates of primary peak locations in the MNI space

#### Time series extraction

After the age, gender, and years of education of all participants were matched, a total of 46 normal subjects (excluding the medial McGurk perceivers) were enrolled into the following analyses. Of note, one subgroup included the strong McGurk perceivers (with a susceptibility > 50%, n = 26), and the other subgroup included the weak McGurk perceivers (with a susceptibility < 50%, n = 20) by referring to (Li et al. 2021). We calculated the first eigenvariates from all voxels in the four ROIs to extract the time series of each participant. Region-specific time series was concatenated over the three sessions (Noppeney et al. 2008) and adjusted to the participant’s F-statistical contrast (effects of interest). In addition, ROIs were activated at the individual level so that the time course could be extracted (*P* < 0.1, uncorrected). As suggested by (Zeidman et al. 2019a), if subjects with no voxel survived in an ROI, we increased the threshold with the step of 0.05 until *P* < 0.5. One participant was excluded because some activations were missed on the single-subject level in ROIs above the predefined threshold. Finally, we included 25 strong and 20 weak perceivers (45 participants in total) for the following analyses. Demographic characteristics are listed in Table 2.

**Table 2:** Characteristics of demographic information

Variables	Strong perceivers (n = 25)	Weak perceivers (n = 20)	*p* value
Gender (Male / Female)	12/13	13/7	0.309^a^
Age (years)	21.84 ± 2.115	20.60 ± 2.257	0.0646^b^
Education (years)	15.40 ± 1.443	15.20 ± 1.824	0.7946^c^
Mean FD	0.088 ± 0.051	0.087 ± 0.040	0.8832^c^
Handedness (Left / Right)	0/25	0/20	

Values are mean ± SD

*FD* frame-wise displacement, *SD* standard deviation

^a^Chi-square test

^b^Two-sample t-test

^c^Mann–Whitney U test

#### Model space construction

A. Endogenous connection (factor 1)

Bidirectional endogenous connections among mSTG, pSTG, and FuG were based on the following findings: (1) the left pSTG is proved to be connected to left mSTG (Friederici et al. 2017) and left FuG (Kreifelts et al. 2007) by some structural data and effective connectivity data; (2) The ROI approach further confirmed that effective connectivity between pSTG and ipsilateral FuG and the effective connectivity between pSTG and mSTG was greatly enhanced during audiovisual integration (Kreifelts et al. 2007); (3) A framework has been established to demonstrate bidirectional connectivity between visual, auditory and multisensory area (Keil and Senkowski, 2018).

Given that the motor cortex was also play an important role in multisensory speech processing (Benoit et al. 2010; Callan et al. 2014; D'Ausilio et al. 2009; Liebenthal and Möttönen 2018; Pulvermuller et al. 2006; Wilson et al. 2004), we further studied the multisensory speech perception processing DCMs that with or without bidirectional connections between PrG and mSTG/pSTG/FuG. To investigate the connection strength differences between groups of strong and weak perceivers, we put forward our hypothesis refer to previous studies (David et al. 2011; Straube et al. 2018). Similar to (Friston et al. 2016), extrinsic connections were modified among different models but respected basic feature and seven families were formed (Supplementary Figure S1 and Table 3): (1) Family 1: PrG bidirectional connected to other three regions; (2) Family 2: PrG bidirectional connected to mSTG and FuG; (3) Family 3: PrG bidirectional connected to pSTG and FuG; (4) Family 4: PrG bidirectional connected to pSTG and mSTG; (5) Family 5: PrG only bidirectional connected to pSTG; (6) Family 6: PrG only bidirectional connected to mSTG; (7) Family 7: PrG only bidirectional connected to FuG. We grouped all three syllable stimuli (/pa/, /ka/ and /ta/) as driving inputs (C-matrix) that entered the system at mSTG and FuG. The C-matrix was identical in all families.

**Table 3:** Model space. 44 unique plausible models were created

Model	Endogenous connection (factor 1)	Modulatory influence (factor 2)	Model	Endogenous connection (factor 1)	Modulatory influence (factor 2)
1	Family 1	a1, b2, c2	23	Family 3	a3, b1, c2
2	Family 1	a2, b2, c2	24	Family 3	a4, b1, c2
3	Family 1	a3, b2, c2	25	Family 3	a1, b1, c3
4	Family 1	a4, b2, c2	26	Family 3	a2, b1, c3
5	Family 1	a1, b3, c2	27	Family 3	a3, b1, c3
6	Family 1	a2, b3, c2	28	Family 3	a4, b1, c3
7	Family 1	a3, b3, c2	29	Family 4	a1, b2, c1
8	Family 1	a4, b3, c2	30	Family 4	a2, b2, c1
9	Family 1	a1, b2, c3	31	Family 4	a3, b2, c1
10	Family 1	a2, b2, c3	32	Family 4	a4, b2, c1
11	Family 1	a3, b2, c3	33	Family 4	a1, b3, c1
12	Family 1	a4, b2, c3	34	Family 4	a2, b3, c1
13	Family 1	a1, b3, c3	35	Family 4	a3, b3, c1
14	Family 1	a2, b3, c3	36	Family 4	a4, b3, c1
15	Family 1	a3, b3, c3	37	Family 5	a1, b1, c1
16	Family 1	a4, b3, c3	38	Family 5	a2, b1, c1
17	Family 2	a1, b2, c2	39	Family 5	a3, b1, c1
18	Family 2	a1, b3, c2	40	Family 5	a4, b1, c1
19	Family 2	a1, b2, c3	41	Family 6	a1, b2, c1
20	Family 2	a1, b3, c3	42	Family 6	a1, b3, c1
21	Family 3	a1, b1, c2	43	Family 7	a1, b1, c2
22	Family 3	a2, b1, c2	44	Family 7	a1, b1, c3

a. The presence or absence of modulation between PrG and pSTG: (1) PrG and pSTG have no modulation. (2) A unidirectional modulation from PrG to pSTG was observed. (3) A unidirectional modulation from pSTG to PrG was observed. (4) A bidirectional modulation was observed between PrG and pSTG

b. The presence or absence of modulation between PrG and mSTG: (1) PrG and mSTG have no modulation. (2) A unidirectional modulation from mSTG to PrG was observed. (3) A bidirectional modulation was observed between PrG and mSTG

c. The presence or absence of modulation between PrG and FuG: (1) PrG and FuG have no modulation between PrG and FuG. (2) A unidirectional modulation from FuG to PrG was observed. (3) A bidirectional modulation was observed between PrG and FuG

2 Modulatory influence (factor 2)

We used all three speech tasks as modulatory inputs (B-matrix), because the single-modality-sourced information is transferred to integration and modulation regions. After the sensory world was sampled using the motor system, selectively extracting the task-relevant information becomes very important due to limited attentional and processing resources (Wolpert et al. 2011). Speech motor control has been proposed to be related to feedback error detection in sensory cortices, which is then projected back to the motor systems to improve the accuracy (Behroozmand et al. 2015). Therefore, we hypothesized that the unidirectional modulation from mSTG to PrG or from FuG to PrG should exist according to the presence of bidirectional intrinsic connection to PrG in our study. Besides, other modulation between PrG and pSTG/mSTG/FuG might exist or not, so differences among different conditions were further analyzed here by modulating all extrinsic connections in each family. Importantly, the factor 2 introduced here varied as a function of the endogenous connection in factor 1. Therefore, this factor was modeled with 10 alternatives and 44 different models were constructed for each participant. The ten different alternatives were as follows:

- The presence or absence of modulation between PrG and pSTG:
  1. PrG and pSTG showed no modulation.
  2. A unidirectional modulation from PrG to pSTG was observed.
  3. A unidirectional modulation from pSTG to PrG was observed.
  4. A bidirectional modulation was observed between PrG and pSTG.
- The presence or absence of modulation between PrG and mSTG:
  1. PrG and mSTG had no modulation.
  2. A unidirectional modulation from mSTG to PrG was observed.
  3. A bidirectional modulation was observed between PrG and mSTG.
- The presence or absence of modulation between PrG and FuG:
  1. PrG and FuG had no modulation between PrG and FuG.
  2. A unidirectional modulation from FuG to PrG was observed.
  3. A bidirectional modulation was observed between PrG and FuG.

Model space visualization was presented in Supplementary Figure S1. For each model, the connectivity parameters (e.g., endogenous connections, modulatory influences, and driving inputs) were estimated using an expectation maximization algorithm (Dempster et al. 1977; Friston 2002).

#### Bayesian model comparison

Given that the subjects included in our study were all healthy volunteers and the characteristics of demographic information were relatively consistent, we first involved all the participants for the model selection. Following model specification and parameter estimation, we performed model comparison across 41 participants with a random-effects (RFX) BMS family level inference procedure, which removes uncertainty about the aspects of model structure other than the characteristic of interest (Penny et al. 2010). The exceedance probability for each family of models was calculated so that the most likely generative family of the observed data could be identified. The family of models with the highest exceedance probability was identified as the “best family of models”. After BMS, we performed a RFX BMA within the best family (Penny et al. 2010; Straube et al. 2018). BMA averaged the connectivity parameters within models of the best family for each participant, weighted by their model exceedance probability. The highest model exceedance probability determined the “winning model” being more fit the observed data, based on which the generated subject-specific connectivity parameters were input into parameter statistics (Parker Jones et al. 2013; Straube et al. 2018; Torrisi et al. 2013). To further identify whether the strong and weak McGurk perceivers have different models with distinct modulatory connections, we performed exploratory analysis by putting the two groups into the modeling separately.

#### Parameter statistics

Classical statistics was used to test the significance of the resulting connection strengths from BMA for both the endogenous connections and modulatory influences (Parker Jones et al. 2013). Prior to classical statistics, we performed multiple linear regression as supported by MATLAB R2018b. We selected age, gender, and years of education to be external regressors to control their effects on connection strengths. Subsequently, we used one-sample *t* test for calculating the significance of each connection within all participants (*n* = 45). In order to investigate the relationship between brain effective connectivity and behavioral performance of McGurk effect, we performed Spearman correlation analysis between regressive connection strengths (A- matrix, B- matrix, A- and B- matrix) and individual McGurk susceptibility. Then, we tested the significance of each connection by using one-sample *t* test within the strong perceivers (*n* = 25) or weak perceivers (*n* = 20). To determine why strong and weak McGurk perceivers have similar neural architecture and even similar network interaction pattern but show different behavioral multisensory illusory perceptions, we adopted two-sample *t* test to evaluate the group differences of each connection. All *p* values were FDR-corrected by using a threshold of *P* < 0.05.

## Results

### DCM model selection

**Fig. 3:**
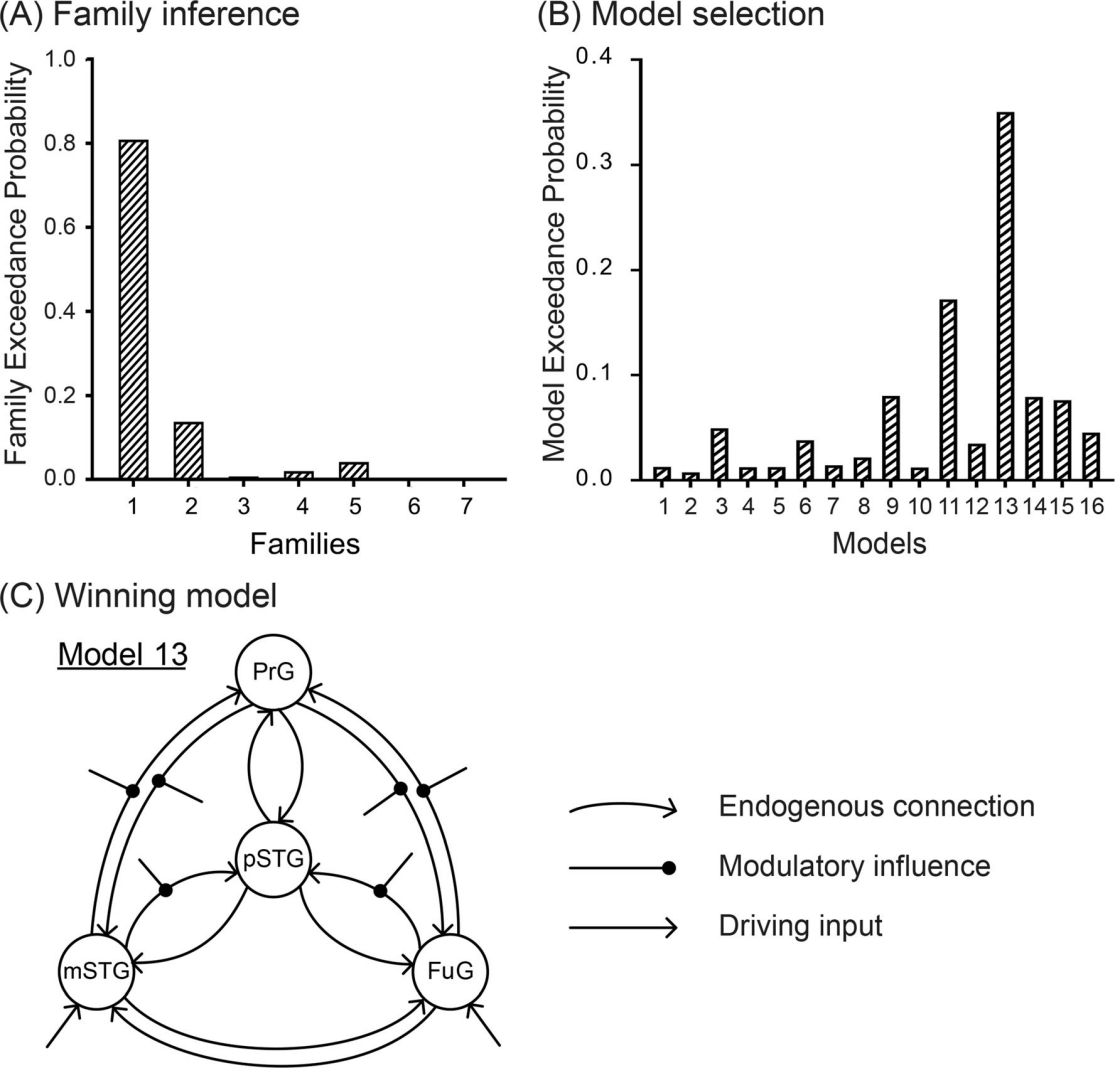
**A** Results of the family level inference in all participants. The best family of models were the DCMs with bidirectional endogenous connections among the four ROIs (Family 1, family exceedance probability = 80.56%). **B** Results from the BMA within family. RFX BMS favored the model with modulatory influences on the connections from PrG to FuG, FuG to PrG, PrG to mSTG, mSTG to PrG, mSTG to pSTG, FuG to pSTG (Model 13, model exceedance probability = 34.92%). (C) Overview of the winning model (Model 13). FuG, fusiform gyrus; pSTG, posterior superior temporal gyrus; mSTG, mid-superior temporal gyrus; PrG, precentral gyrus

RFX BMS provided evidence for the “best family of models” to be the generative models for the observed data. Family-level inference revealed that the models with bidirectional endogenous connections between the four regions (Family 1) outperformed all families (family exceedance probability = 80.56%, Fig. 3A). Among the 16 “best family of models”, the model with modulatory influences on the connections from PrG to FuG, from FuG to PrG, from PrG to mSTG, from mSTG to PrG, from mSTG to pSTG, from FuG to pSTG (model 13) won over 15 other models (Model Exceedance Probability = 34.92%, Fig. 3B). An overview of the winning model is illustrated in Fig. 3C. Exploratory analysis presented that the strong and weak McGurk have the same best family (family 1) but have different models with several distinct modulatory connections. Specifically, the RFX BMS favored the Model 13 in the strong McGurk perceivers, while Model 11 in the weak McGurk perceivers (Supplementary Figures S2-S3).

### DCM parameter statistics

**Fig. 4:**
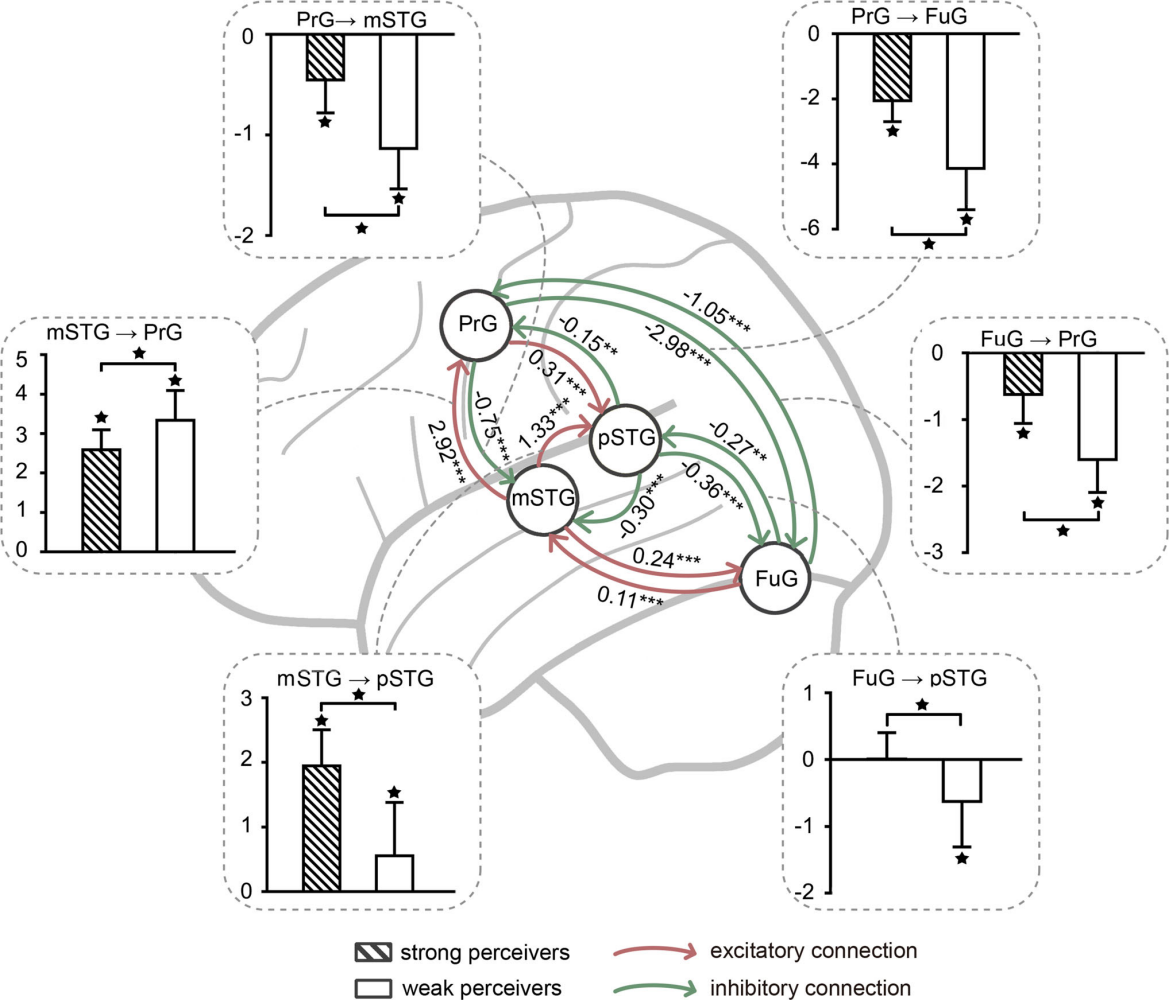
Winning model with averaged coupling strength (A- and B- matrix) for excitatory (red) and inhibitory (green) connections (*P < 0.05, **P < 0.01, ***P < 0.001, uncorrected). Coupling strength differences between strong and weak perceivers are illustrated using bar plots (solid star: *P* < 0.05, FDR-corrected). FuG, fusiform gyrus; pSTG, posterior superior temporal gyrus; mSTG, mid-superior temporal gyrus; PrG, precentral gyrus

The DCM parameter statistics are summarized in Table 4 and Fig. 4. Based on the winning model (model 13), the endogenous connections from mSTG to PrG (*P* = 0.857) and the modulatory connections from FuG to pSTG (0.352) failed to reach the statistical significance level and the remaining connections were all significant (Table 4, < 0.05, FDR-corrected). The averaged coupling strength (A- and B- matrix) showed excitatory connections (shown as red edges in Fig. 4) from mSTG to PrG and pSTG. The remaining coupling connections were inhibitory connections (green edges in Fig. 4). Regarding brain effective connectivity and individual behavioral performance of McGurk effect, we found that the individual McGurk susceptibility is correlated with the coupling connection strengths (FDR corrected, *P* < 0.05). To further test the coupling strength differences between strong and weak perceivers, we compared these six connections (mSTG → PrG, mSTG → pSTG, PrG → mSTG, PrG → FuG, FuG → PrG, FuG → pSTG) with modulatory influences (B matrix) (see bar plots in Fig. 4).

**Table 4:** DCM parameters and statistics

Connection	(n = 45)	All participants						Strong perceivers (n = 25)				Weak perceivers (n = 20)				Group differences	
		Strength		Statistics		Correlation		Strength		Statistics		Strength (Hz)		Statistics		Statistics	
From	To	Mean	SD	*t*_(44)_	*p*	r	*P*-value	Mean	SD	*t*_(24)_	*P*	Mean	SD	*t*_(19)_	*P*	*t*_(43)_	*P*
Endogenous connections (A matrix)																	
PrG	pSTG	0.31	0.11	18.03	**< 0.001**	0.54	**< 0.001**	0.34	0.07	24.11	**< 0.001**	0.26	0.14	8.26	**< 0.001**	2.28	0.028
PrG	mSTG	− 0.32	0.22	− 9.57	**< 0.001**	− 0.77	**< 0.001**	− 0.49	0.13	− 18.21	**< 0.001**	− 0.11	0.08	− 5.62	**< 0.001**	− 11.10	**< 0.001**
PrG	FuG	0.12	0.16	5.25	**< 0.001**	0.37	**0.01**	0.17	0.18	4.88	**< 0.001**	0.06	0.09	2.77	**0.01**	2.62	0.012
pSTG	PrG	− 0.15	0.36	− 2.71	**0.010**	− 0.74	**< 0.001**	− 0.44	0.18	− 12.40	**< 0.001**	0.22	0.10	9.47	**< 0.001**	− 14.72	**< 0.001**
pSTG	mSTG	− 0.30	0.34	− 5.81	**< 0.001**	− 0.76	**< 0.001**	− 0.56	0.20	− 13.80	**< 0.001**	0.03	0.15	0.75	0.46	− 10.66	**< 0.001**
pSTG	FuG	− 0.36	0.29	− 8.47	**< 0.001**	− 0.61	**< 0.001**	− 0.53	0.21	− 12.52	**< 0.001**	− 0.15	0.21	− 3.09	**< 0.001**	− 6.09	**< 0.001**
mSTG	PrG	0.01	0.36	0.18	0.857	0.81	**< 0.001**	0.31	0.12	12.89	**< 0.001**	− 0.37	0.12	− 13.88	**< 0.001**	18.90	**< 0.001**
mSTG	pSTG	0.36	0.16	14.95	**< 0.001**	− 0.47	**0.001**	0.28	0.14	10.15	**< 0.001**	0.46	0.13	15.80	**< 0.001**	− 4.53	**< 0.001**
mSTG	FuG	0.24	0.45	3.66	**0.001**	− 0.71	**< 0.001**	− 0.13	0.15	− 4.17	**< 0.001**	0.70	0.17	18.43	**< 0.001**	− 17.29	**< 0.001**
FuG	PrG	− 0.35	0.18	− 13.15	**< 0.001**	0.63	**< 0.001**	− 0.25	0.16	− 8.05	**< 0.001**	− 0.48	0.12	− 18.28	**< 0.001**	5.42	**< 0.001**
FuG	pSTG	− 0.35	0.16	− 14.40	**< 0.001**	0.17	0.26	− 0.32	0.18	− 8.87	**< 0.001**	− 0.38	0.13	− 12.95	**< 0.001**	1.23	0.225
FuG	mSTG	0.11	0.13	5.38	**< 0.001**	0.19	0.21	0.13	0.15	4.18	**< 0.001**	0.08	0.11	3.46	**< 0.001**	1.06	0.297
Modulatory influences (B matrix)																	
PrG	mSTG	− 0.44	0.66	− 4.44	**< 0.001**	0.64	**< 0.001**	0.04	0.37	0.48	0.635	− 1.03	0.42	− 11.00	**< 0.001**	9.03	**< 0.001**
PrG	FuG	− 3.10	1.36	− 15.35	**< 0.001**	0.67	**< 0.001**	− 2.23	0.66	− 17.01	**< 0.001**	− 4.20	1.20	− 15.58	**< 0.001**	6.99	**< 0.001**
mSTG	PrG	2.91	0.94	20.87	**< 0.001**	− 0.59	**< 0.001**	2.28	0.48	23.46	**< 0.001**	3.71	0.73	22.81	**< 0.001**	− 7.91	**< 0.001**
mSTG	pSTG	0.97	1.02	6.37	**< 0.001**	0.69	**< 0.001**	1.67	0.55	15.27	**< 0.001**	0.10	0.78	0.57	0.58	7.94	**< 0.001**
FuG	PrG	− 0.70	0.55	− 8.59	**< 0.001**	0.67	**< 0.001**	− 0.37	0.37	− 4.90	**< 0.001**	− 1.12	0.43	− 11.59	**< 0.001**	6.25	**< 0.001**
FuG	pSTG	0.08	0.56	0.94	0.352	0.66	**< 0.001**	0.33	0.26	6.43	**< 0.001**	− 0.24	0.67	− 1.60	0.13	3.94	**< 0.001**
Resulting coupling strength (A + B)																	
PrG	mSTG	− 0.75	0.50	− 10.19	**< 0.001**	0.54	**< 0.001**	− 0.45	0.33	− 6.88	**< 0.001**	− 1.13	0.40	− 12.59	**< 0.001**	6.28	**< 0.001**
PrG	FuG	− 2.98	1.38	− 14.47	**< 0.001**	0.66	**< 0.001**	− 2.05	0.64	− 16.03	**< 0.001**	− 4.14	1.17	− 15.84	**< 0.001**	7.61	**< 0.001**
mSTG	PrG	2.92	0.73	26.89	**< 0.001**	− 0.30	0.04	2.59	0.51	25.40	**< 0.001**	3.34	0.76	19.70	**< 0.001**	− 3.96	**< 0.001**
mSTG	pSTG	1.33	0.98	9.13	**< 0.001**	0.67	**< 0.001**	1.95	0.56	17.36	**< 0.001**	0.56	0.83	3.01	**0.01**	6.70	**< 0.001**
FuG	PrG	− 1.05	0.67	− 10.52	**< 0.001**	0.70	**< 0.001**	− 0.62	0.44	− 7.09	**< 0.001**	− 1.60	0.50	− 14.43	**< 0.001**	7.04	**< 0.001**
FuG	pSTG	− 0.27	0.62	− 2.94	**0.005**	0.56	**< 0.001**	0.01	0.40	0.10	0.920	− 0.62	0.68	− 4.09	**< 0.001**	3.89	**< 0.001**

Significant *p* values (*P* < 0.05, FDR-corrected) are rendered in bold

*SD* standard deviation, *FuG* fusiform gyrus, *pSTG* posterior superior temporal gyrus, *mSTG* mid-superior temporal gyrus, *PrG* precentral gyrus

#### Connection from PrG to mSTG and that from PrG to FuG

The connection from PrG to mSTG and FuG showed a significant inhibitory effect (*P* < 0.001). In addition, the greater McGurk susceptibility, the stronger the network coupling including PrG → mSTG (*r* = 0.54, *P* < 0.001), PrG → FuG (*r* = 0.66, *P* < 0.001). By contrast, strong perceivers showed a significant lower inhibitory effect in both connections (*P* < 0.001).

#### Connection from mSTG/FuG to PrG

The connection from mSTG to PrG showed a significant excitatory effect (*P* < 0.001), and the correlation between that and McGurk susceptibility reached the trend level (*r* = − 0.30, *P* = 0.04). The excitatory effect of strong perceivers was significantly lower (*P* < 0.001). Moreover, an inhibitory connection was observed from FuG to PrG (*P* < 0.001), which was positive correlated with McGurk susceptibility (*r* = 0.70, *P* < 0.001). The inhibitory effect is significantly lower in strong perceivers (*P* < 0.001).

#### Connection from mSTG/FuG to pSTG

The connection from mSTG to pSTG showed a significant excitatory effect (*P* < 0.001), and the connection is positive correlated with McGurk susceptibility (*r* = 0.67, *P* < 0.001). The higher coupling was observed in strong perceivers (*P* < 0.001). In terms of the connection from FuG to pSTG, an inhibitory connection was observed (*P* = 0.005), which is positive correlated with McGurk susceptibility (*r* = 0.56, *P* < 0.001). Besides, the group comparison showed significant less inhibition in strong perceivers (*P* < 0.001).

## Discussion

We investigated how motor cortex and pSTG dynamically interact with the primary sensory inputs during the multisensory speech perception. Behavioral and fMRI audiovisual experiments were carried out, and DCM analysis was applied to reveal the effective connectivity between the cross-modal areas of PrG and pSTG and the unisensory cortices of mSTG and FuG. Our results favored a fully connected model showing that both PrG and pSTG enhanced the auditory signal processing but suppressed the visual signal influence. This observation suggests that multisensory speech perception highly rely on interactions of auditory stream to motor cortex and pSTG. More importantly, the coupling strength of the connections associated with PrG and pSTG was correlated with individual behavioral McGurk susceptibility. Additionally, between-group coupling strength statistics showed that compared with the weak McGurk effect perceivers, the strong perceivers processed less inhibitory visual inputs and excitatory auditory information in PrG and integrated more audiovisual contents in pSTG. Together, these results suggest that the PrG and pSTG interact dynamically with the primary cortices during audiovisual speech and prove that the motor cortex plays a specific role in adjusting the gain and salience between the auditory and visual modalities.

It is helpful to get new mechanistic insights on the functional roles of the PrG and STG/S during the multisensory speech processing from the classical DCM analysis. Our results favored a winning family with a bidirectional endogenous connection from PrG to pSTG, mSTG, and FuG, which are consistent with the previous literature supporting the involvement of motor cortex in audiovisual tasks (Benoit et al. 2010; Callan et al. 2014; D'Ausilio et al. 2009; Liebenthal and Möttönen 2018; Pulvermuller et al. 2006; Skipper et al. 2005; Wilson et al. 2004). Furthermore, we found the winning model in family 1 with the modulations of input on the connections from PrG to mSTG, PrG to FuG, mSTG to PrG, FuG to PrG, mSTG to pSTG, and FuG to pSTG (model 13). The observed dynamic processes suggest that pSTG and motor cortex both serve as important neural substrates in multisensory speech perception. Aside from emphasizing the importance of both mSTG and PrG in multimodal perceptual processes, our winning model show potential different functions between the above two brain areas. In specific, pSTG was only subjected to the influences from bottom-up sensory inputs, while PrG generated top-down feedback modulation while receiving the information flow. Meanwhile, a recent study has proposed that distinct mechanisms may occur in these two regions, in which the STG is likely to exhibit auditory and visual inputs redundantly, while PrG represents the sensory inputs synergistically (Park et al. 2018). As a classic multi-sensory integration area, the pSTG has been proved, in many studies on functional neuroimaging, to be associated with audiovisual speech integration (Daniel et al. 2004; Michael et al. 2004). A recent DCM study has shown that STS could receive and reorder the speech and then determined the multimodal syllable representation (Bouton et al. 2020). In contrast, PrG has been adopted as the top-down modulator to facilitate audiovisual speech comprehension and multisensory integration (Choi et al. 2018; Park et al. 2018). In other words, auditory and visual inputs may be processed at varying degrees of integration in pSTG, and as a modulator, the PrG may require more information from sensory areas. Our results support that the auditory and visual feedforward and feedback interactions may be facilitated by the modulation through PrG, which is helpful to calibrate and characterize the multi-sensory information accurately. The speech perception motor theory has been proved to support the regulation of motor cortex (Corballis 2010), in which the motor cortex involves in audiovisual mapping the sensory inputs onto matching motor representations. Therefore, our DCM findings support the notion that it is possible distinct brain circuits (such as PrG and pSTG) for parallel sensory processing and eventually form a multisensory percept based on their interactive cerebral connections.

In addition, significant positive correlations were found between individual McGurk susceptibility and coupling strengths including PrG → mSTG, PrG → FuG, FuG → pSTG, mSTG → pSTG, and FuG → PrG. Whereas, slight negative correlation (uncorrected) was found between McGurk susceptibility and the coupling strength of connection from mSTG to PrG. In other words, subjects with stronger behavioral McGurk effect were more likely to have higher coupling connectivity from FuG to PrG, or lower coupling connectivity from mSTG to PrG, which provides further evidence that the PrG modulates more visual information relative to auditory information in the population who are more likely to perceive the McGurk effect. These correlation results suggest that the modulatory process in the motor cortex, as well as the audiovisual information integration in the pSTG play important roles in McGurk’s illusory behavior.

Regarding the different behavioral McGurk illusory susceptibility in the healthy population, we further explore the group differences in effective connectivity. Firstly, by putting all the subjects into the modeling, between-group statistical and post-hoc analyses suggested that the mean coupling strength of the winning model (model 13) differed significantly between strong and weak perceivers, including the connection from mSTG to pSTG and that from FuG to pSTG. The connection from mSTG to pSTG is excitatory, whereas that from FuG to pSTG is inhibitory in both groups. In addition, strong perceivers showed more excitatory auditory signal information and less inhibitory visual information flow to pSTG. It seems that the diverse dependency level of inhibitory visual input in pSTG may be the key aspect for varying individual audiovisual susceptibility. Evidence have shown that patients with schizophrenia revealed significantly reduced connectivity in the verbal pathway (from left middle temporal gyrus to left STS) (Wroblewski et al. 2020). Furthermore, the relationship between the McGurk effect and Odd or Eccentric Behaviour associated with schizotypal personality traits was fully mediated by visual accuracy (Muller et al. 2021). These can be considered as a stronger focus on visual information instead of auditory information in the integration process of speech. Interestingly, people relying more on auditory modal in daily (e.g., musicians) are likely to be weak McGurk perceivers (Proverbio et al. 2016). Significant differences were observed in the coupling connectivity from primary sensory areas (mSTG, FuG) to PrG between the McGurk perceiver subgroups. We observed less excitatory auditory streams and inhibitory visual streams flowed into PrG in strong perceivers. Additionally, significant differences were observed in the coupling connectivity from PrG to primary sensory areas (mSTG, FuG) between the two subgroups. The feedback error detection in sensory cortices is involved by the speech motor control, based on which the motor-related areas were activated to adjust (modulate) the parameters during the speech perception (Behroozmand et al. 2015). Prior combined TMS and fMRI study has demonstrated that disruption of the motor cortex lip areas effectively reduced the McGurk effect, indicating that the motor cortex contributes to the detection and resolution of multisensory incompatibility and participates in regulating the speech perception (Murakami et al. 2018). Based on the potential regulation processes in the motor cortex for adjusting the gain and salience between auditory and visual modalities, we speculated that stronger McGurk perceivers may consider that the visual channel is more reliable than the auditory channel. Our such results are consistent with previous findings that incoherent audiovisual context would decrease the weight of visual stream (Nahorna et al. 2012, 2015).

It is worth noting that the strong and weak subgroups have several different modulatory connections when they were put into the modeling separately: the connection from pSTG to PrG in weak group and that from PrG to mSTG in strong group. It is possible that the strong McGurk perceivers showed more downstream from PrG to pSTG. Moreover, the weak McGurk perceivers may be more effective in processing multisensory information, given that the integration area of pSTG would provide feedforward effect to the motor area. Such finding described here is consistent with the ideas in several previous studies, showing that the specificity of phoneme representations and the network connectivity of dorsal stream can be enhanced by lip movement, improving the speech perception (Zhang and Du 2022). It maybe relate to speech processing dorsal stream in sensorimotor integration where the pSTG functions as a sensorimotor interface while the PrG provides articulatory predictions of upcoming speech from other modalities (e.g., lip movements) and feedback to the pSTG for integrating top-down prediction and bottom-up speech (Du et al. 2014; Hickok and Poeppel 2007).

The current study is subjected to several limitations. First, illusion rate of the sample included was 40–60%. To further confirm the findings and accurately follow the brain modifications, researchers should expand the recruitment for large cohorts of participants. Second, mechanisms for processing the McGurk audiovisual pairings differs from that processing the natural audiovisual speech events, but the McGurk pairs were not performed in this study. Therefore, we will design more experiment for congruent and incongruent audiovisual stimuli. Third, whether fMRI signals are conductive to casual inference remain inconclusive, because the accuracy of causal modeling with fMRI data is adversely affected by limited time (Ramsey et al. 2010). Fourth, the individual connections strength is subjected to a certain variability, that is, parameters represented by their covariance matrix are uncertain, so further studies especially in clinical cohorts, might be extended to test the alterations in effective connectivity during multisensory speech perception due to pathological factors (e.g., schizophrenia) (Lu and Pan 2020), or brain lesion (e.g., aphasia) (Krason et al. 2022) by using Parametric Empirical Bayes analyses (PEB) (Bencivenga et al. 2021; Friston et al. 2016; Zeidman et al. 2019a, 2019b). Finally, the current study included non-computer-generated stimuli. In future research, a new informative approach should be employed to investigate the “bistable” support of false audiovisual speech perception induced by audiovisual integration (Thézé et al. 2020).

In summary, our study supports a new perspective on multisensory speech processing, which considers the auditory and visual information integration of pSTG and the characterization of the functional modulation of PrG. Furthermore, we demonstrate that the modulatory process in the motor cortex, as well as the audiovisual information integration in the pSTG play important roles in McGurk’s illusory behavior. Our findings unveil the dynamic interactive processes among cross-modal regions and unisensory cortices during the multisensory speech processing and particularly highlight the specific function of motor cortex in modulating the gain and salience between auditory and visual modalities.

### Supplementary Information

Below is the link to the electronic supplementary material.

Supplementary file1 (DOCX 1110 kb)

## Data Availability

The data that support the findings of this study are available from the corresponding author upon reasonable request.
