# Supplementary material for: Dynamic causal modeling analysis reveals the modulation of motor cortex and integration in superior temporal gyrus during multisensory speech perception
Source: Cogn Neurodyn. 2023 Mar 4;18(3):931–46. doi: 10.1007/s11571-023-09945-z (PMC11143173; doi:10.1007/s11571-023-09945-z)
Supplement: Supplementary file 1 — Supplementary file1 (DOCX 1110 kb) [file 11571_2023_9945_MOESM1_ESM.docx]

**Dynamic causal modeling analysis reveals the modulation of motor cortex and integration in superior temporal gyrus during multisensory speech perception**

Abbreviated title: Dynamic interactions in multisensory speech processing

Ting Zou^1^, Liyuan Li^1^, Xinju Huang^1^, Chijun Deng, Xuyang Wang, Qing Gao^1^, Huafu Chen^1*^, and Rong Li^1*^

^1^ The Clinical Hospital of Chengdu Brain Science Institute, MOE Key Laboratory for Neuroinformation, High-Field Magnetic Resonance Brain Imaging Key Laboratory of Sichuan Province, School of Life Science and Technology, University of Electronic Science and Technology of China, Chengdu, 610054, P.R. China.

*Correspondence to: Rong Li, Email: [rongli1120@gmail.com](mailto:rongli1120@gmail.com);

Huafu Chen, Email: chenhf@uestc.edu.cn;

Supplementary materials

**Supplementary Table S1 Brain regions with signifcant diferences of fMRI activation between strong and weak perceivers**

| Cluster number | Brain regions | MNI coordinates  (x, y, z) | Cluster size | *T*-value |
| --- | --- | --- | --- | --- |
| Cluster 1 | L Cerebellum | 0 -36 -48 | 783 | 4.14 |
| Cluster 2 | L Middle temporal gyrus | -60 -51 21 | 1474 | 4.76 |
|  | L Superior temporal gyrus | -48 -45 18 |  | 3.66 |
|  | L Precentral gyrus | -48 3 30 |  | 2.7 |
|  | L Fusiform gyrus | -36 -15 -27 |  | 3.13 |
| Cluster 3 | R Parahippocampal gyrus | 15 -9 -21 | 166 | 2.91 |
| Cluster 4 | L Inferior temporal gyrus | -51 -48 -12 | 97 | 2.66 |
| Cluster 5 | R Inferior orbitofrontal frontal gurus | 48 21 -6 | 504 | 3.13 |
| Cluster 6 | R Middle temporal gyrus | 57 -66 6 | 378 | 2.83 |
| Cluster 7 | R Inferior frontal gyrus, triangular part | 33 15 27 | 119 | 2.89 |
| Cluster 8 | L Middle occipital lobe | -33 -90 27 | 137 | 3.16 |
| Cluster 9 | R Superior parietal lobe | 33 -51 69 | 105 | 2.42 |
| Cluster 10 | L Supplementary motor area | -3 12 72 | 546 | 3.35 |
| Cluster 11 | L Superior frontal gyrus | 15 -12 72 | 121 | 3.10 |

All the clusters survived by using a threshold of p<0.05 and extent threshold k=20 voxels. MNI Montreal Neurological Institute; X, Y, Z coordinates of primary peak locations in the MNI space


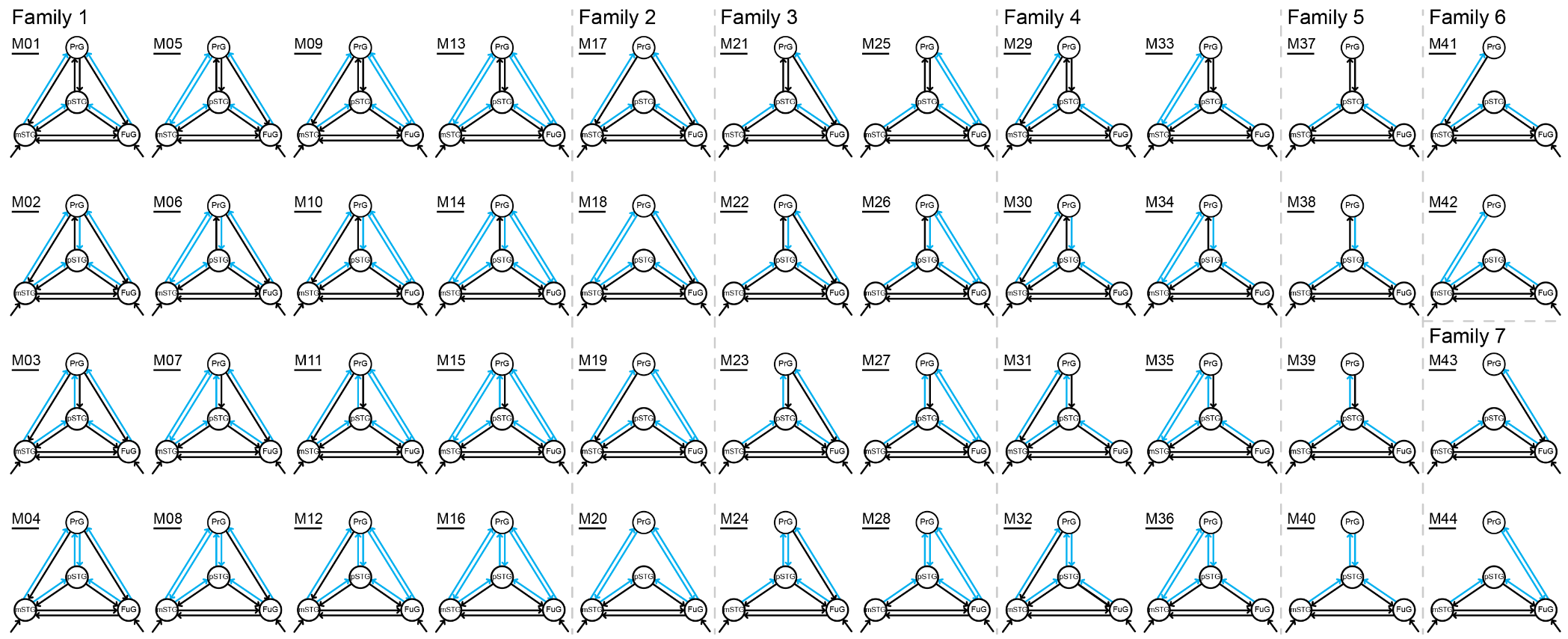


**Supplementary Figure S1. Model Space visualization.** Modulatory influence of task was showed in blue.

**
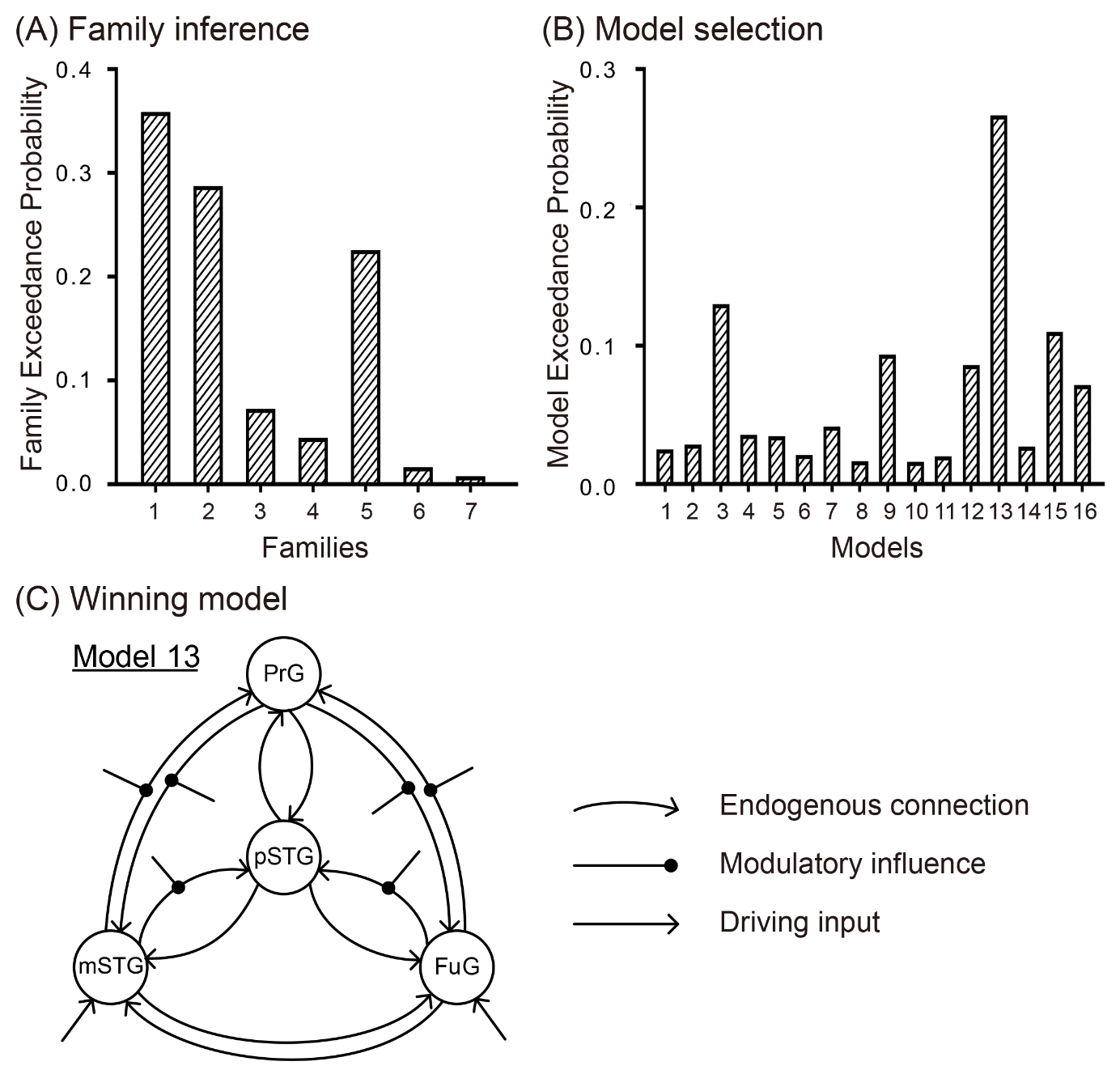
**

**Supplementary Figure S2.** (A) Results of the family level inference in strong McGurk effect perceivers (n = 25). The best family of models are the DCMs with bidirectional endogenous connections between the four ROIs (Family 1, family exceedance probability = 35.67%). (B) Results from the Bayesian Model Averaging within family. Random-effects Bayesian Model Selection favored the model with modulatory influences on the connections from PrG to FuG, FuG to PrG, mSTG to pSTG, FuG to pSTG, PrG to mSTG, mSTG to PrG (Model 13, model exceedance probability = 26.53%). (C) Overview of the winning model (Model 13). PrG, precentral gyrus; mSTG, mid-superior temporal gyrus; pSTG, posterior superior temporal gyrus; FuG, fusiform gyrus.


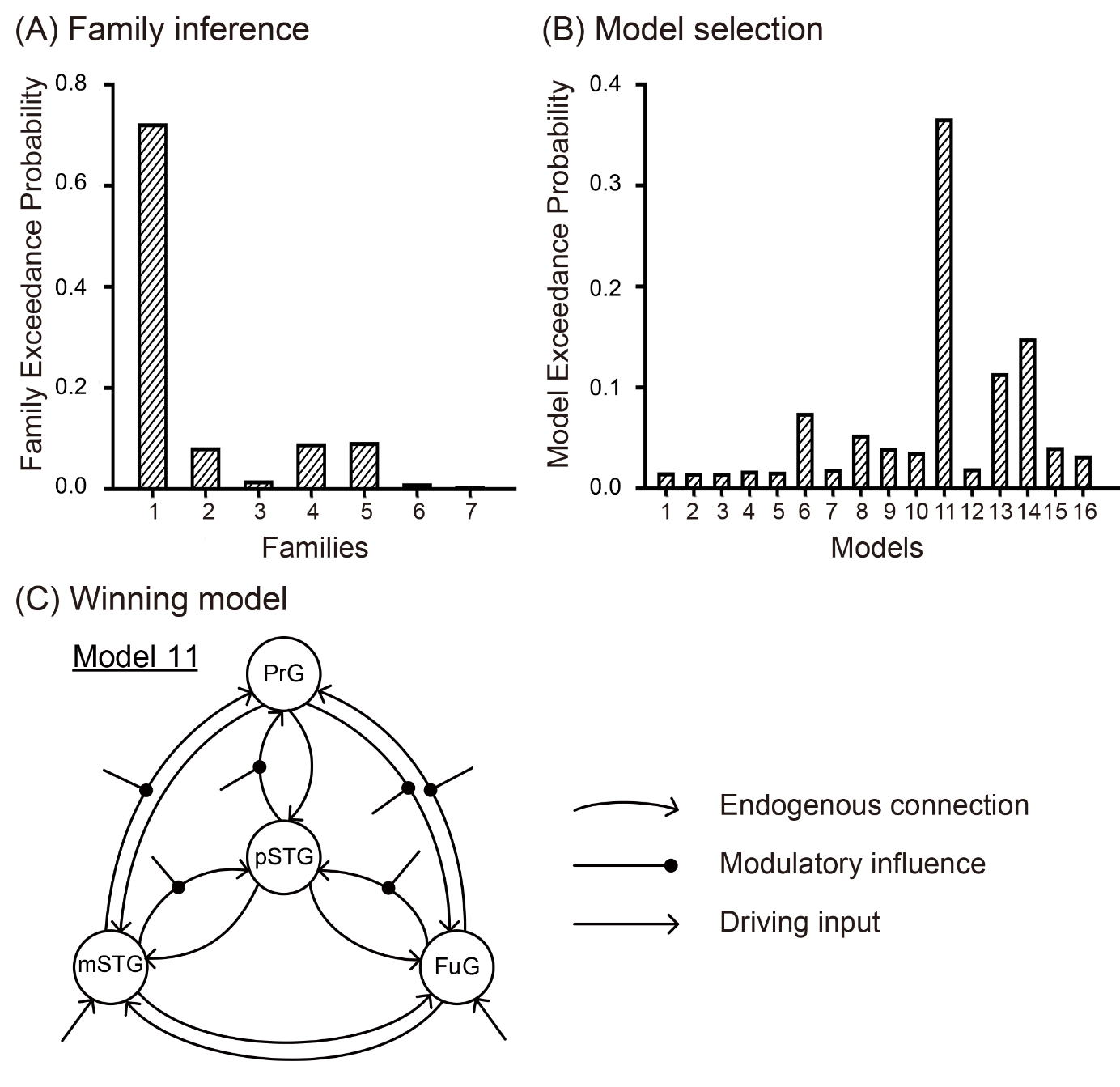


**Supplementary Figure S3.** (A) Results of the family level inference in all participants in week McGurk effect perceivers (n = 20). The best family of models are the DCMs with bidirectional endogenous connections between the four ROIs (Family 1, family exceedance probability = 71.97%). (B) Results from the Bayesian Model Averaging within family. Random-effects Bayesian Model Selection favored the model with modulatory influences on the connections from PrG to FuG, pSTG to PrG, mSTG to PrG, mSTG to pSTG, FuG to PrG, and FuG to pSTG (Model 11, model exceedance probability = 36.44%). (C) Overview of the winning model (Model 11). PrG, precentral gyrus; mSTG, mid-superior temporal gyrus; pSTG, posterior superior temporal gyrus; FuG, fusiform gyrus.
